# Substrate Impact on MR Characteristics of Carbon Nano Films Explored via AFM and Raman Analysis

**DOI:** 10.3390/ma14133649

**Published:** 2021-06-30

**Authors:** Awais Siddique Saleemi, Muhammad Hafeez, Muhammad Saeed, Ali Abdullah, Muhammad Anis-ur- Rehman, Shern-Long Lee

**Affiliations:** 1 Institute for Advanced Study, Shenzhen University, Shenzhen 518060, China; assaleemi@szu.edu.cn; 2 Key Laboratory of Optoelectronic Devices and Systems of Ministry of Education and Guangdong Province, College of Optoelectronic Engineering, Shenzhen University, Shenzhen 518060, China; mhafeez007@yahoo.com; 3 Department of Physics, Lahore Garrison University, Lahore 54761, Pakistan; 4 College of Nuclear Science and Engineering, East China University of Technology, Nanchang 330013, China; saeedphysics96@gmail.com; 5 School of Science, University of Management and Technology, Sialkot 51310, Pakistan; ali.abdullah@skt.umt.edu.pk; 6 Applied Thermal Physics Laboratory, Department of Physics, COMSATS University, Islamabad 44000, Pakistan; marehman@comsats.edu.pk

**Keywords:** amorphous carbon, disorder degree, magnetoresistance, magnetic sensors

## Abstract

Recent advances in the fabrication and classification of amorphous carbon (a-Carbon) thin films play an active part in the field of surface materials science. In this paper, a pulsed laser deposition (PLD) technique through controlling experimental parameters, including deposition time/temperature and laser energy/frequency, has been employed to examine the substrate effect of amorphous carbon thin film fabrication over SiO_2_ and glass substrates. In this paper, we have examined the structural and magnetoresistance (MR) properties of these thin films. The intensity ratio of the G-band and D-band (I_D_/I_G_) were 1.1 and 2.4, where the C(*sp*^2^) atomic ratio for the thin films samples that were prepared on glass and SiO_2_ substrates, were observed as 65% and 85%, respectively. The MR properties were examined under a magnetic field ranging from −9 T to 9 T within a 2-K to 40-K temperature range. A positive MR value of 15% was examined at a low temperature of 2 K for the thin films grown on SiO_2_ substrate at a growth temperature of 400 °C using a 300 mJ/pulse laser frequency. The structural changes may tune the magnetoresistance properties of these a-Carbon materials. These results were demonstrated to be highly promising for carbon-based spintronics and magnetic sensors.

## 1. Introduction

In the past two and half decades, since graphene was determined to be a “wonder material”, conjugated carbon-based materials, especially carbon nanomaterials and fullerenes, have been considered as important research due to their exclusive properties. Among the carbon allotropes, due to tremendous findings, for example gas sensing, magnetic sensing, solar cells, and opto-electronics, amorphous carbon (a-Carbon) thin films have attracted special attention [1,2,3,4,5]. The magnetotransport characteristics of a-Carbon thin films largely depend on the size of C(*sp*^2^) clusters, structural disorder, and C(*sp*^2^) fractions. Disorder is an important factor that influences the conductivity of materials. Therefore, most scientists have seriously investigated the relationship between magnetotransport properties at structural disorders, and they have also examined the underpinning mechanisms of the transport properties of pure a-Carbon materials. Although an enormous amount of work has been performed, numerous hidden transport properties of these films remain essential to uncover [6,7,8,9,10].

The commercialization of a-Carbon materials in solar-cell applications is mostly hindered by challenges, such as technical hitches in governing the density of intrinsic defects and in adjusting the conduction type and carrier density of the a-Carbon materials. In this regard, numerous elements, for example phosphorus, boron, sulfur, and nitrogen, have been exploited as dopants in a-Carbon materials. Amongst these elements, nitrogen and phosphorus have been proven to be n-type dopants, whereas boron is considered a p-type one. Only boron has been observed to be a p-type dopant in a-Carbon materials [11,12,13,14,15]. Hence, we only had boron as an option for p-type doping in a-Carbon materials, although its activation energy (about 200–300 meV) was comparatively higher. The use of boron as a dopant might not only influence the conduction type and carrier density, but may also modulate the band gap of a-Carbon materials. However, this interaction largely causes an inconvenience in monitoring the properties of a-Carbon materials. Therefore, there is a strong need to examine the latest dopants, particularly p-type dopants having lower activation energy and minor effects on the band structure of a-Carbon materials [7,15,16,17].

Other than doped a-Carbon, there are many uses for un-doped a-Carbon, such as its contributions to the undeviating impact on the amorphous nature and magnetotransport properties of a-Carbon. Intensive studies of doped a-Carbon magnetotransport properties have already done in previous years; however, un-doped amorphous carbon studies are rare, which have specific significance [13,18,19,20,21]. Sagar et al. reported on CVD-grown a-Carbon with a negative MR magnitude of 2.7% and an angular MR of 9.5%. Due to the low band gap value of 0.6 eV, the un-doped non-crystalline a-Carbon is a very poor semiconductor, which makes it a weak contender for many optoelectronic applications [22,23]. The structure of a-Carbon has substantial consequences in its magnetotransport uses, since a-Carbon involves a short-range ordered structure rather than long-range ordered structures [24].

Subsequently, we have recently performed a series of experiments, in which we have reported on a-Carbon structure-dependent MR properties, and we reported an enhanced angular MR of 18% and negative MR of 13% for a-Carbon thin films fabricated through a CVD technique. Furthermore the mechanism of such MR properties was investigated and it was found that they greatly depend on grain boundary scattering (GBS) and weak localization theory (WLT) [25,26]. Furthermore, the correlation of MR of a-Carbon with spintronics, has greatly demanded attention in recent years owing to its magnetoresistance applications; for higher magnetic fields, a-Carbon MR is still considered as unsaturated. Afterwards, a-Carbon thin films were synthesized on a glass substrate using a PLD technique and we observed a very large positive MR. The Efros–Shklovskii variable range hopping model has been anticipated to be a suitable transport mechanism for these thin films [27].

In this paper, we aimed to report on the substrate effect on the structural and magnetotransport properties of a-Carbon thin films fabricated using a PLD technique to control different growth parameters. Furthermore, the prepared thin films have been characterized using numerous analytical tools. We have investigated the enormous variations in the structure and MR of a-Carbon samples due to the glass and SiO_2_ substrate, respectively. To determine the structural growth of the thin films, the substrate plays a significant role, which may tune the magnetoresistance properties.

## 2. Experimental Method

The a-Carbon thin films were fabricated using a pulsed laser deposition (PLD) technique. Silicon dioxide wafer and glass substrates with a 1-mm thickness were used for the fabrication of a-Carbon film samples. We cleaned the substrates via sonication with ethanol and acetone, and surface contaminations were removed by a rinsing process using deionized water.

First, pure graphite (99.9%) powder was used in the preparation of the target. This powder was cold pressed into a cylindrical target at 50 MPa. Then, target sintering was performed at 1300 °C for 30 min. Subsequently, the target and substrates were set in the PLD chamber for thin-film deposition. Subsequently, the chamber was pumped to 2.5 × 10^−4^ Pa. The substrates were heated up to a temperature of 400 °C. Then, a 300 mJ/pulse laser energy was used to bombard the carbon target for 15 min. Then, the as-grown a-Carbon samples were annealed for 30 min. These samples were then used for further characterization using a series of surface analytical tools. 

The magnetotransport properties and resistivity measurements were done through a physical-property measurement system, from 2 K to 300 K, and under a magnetic field of −9~9 T. The indium pressing method was used with a mask size of ~1 mm^2^ for the preparation of the metal electrodes. The total sample length and width was ~10 mm and ~5 mm, respectively, while the size of electrode I was ~1 mm^2^ and the distance between the contacts (L) was ~2 mm.

The Lambda PhysicK (LPX-250) system was used for PLD. Renishaw InVia Raman spectroscopy was then used for measuring the excitation wavelength of 514 nm. With the help of high-resolution transmission electron microscopy (HRTEM) the structure of a-Carbon thin films was examined.

## 3. Results and Discussion

### 3.1. Structural Analysis

In the current work, different growth parameters and changes in substrate materials were used to observe the changes in the structural qualities of a-Carbon thin films. These structural features are supposed to influence the magnetoresistance properties. To investigate the structural changes, different types of analytical tools were used for the quantitative measurements of the structural parameters. The behavior of the thin films and the randomly oriented graphitic crystals on their surfaces were investigated by HRTEM. For the qualitative measurement of the nature of the amorphous thin films, X-ray diffraction was used. Raman and XPS were used to explore the quantitative structural constraints, such as the G-band, D-band, FWHM of D-band, and the C(*sp*^3^) and C(*sp*^2^) content.

**Figure 1:**
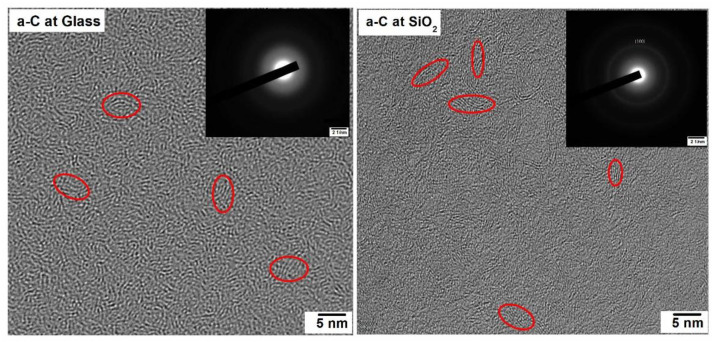
HRTEM of sample thin films; inset: diffraction pattern.

With the help of HRTEM, the structure of the a-Carbon films was examined. We examined the randomly oriented graphitic structures with a size of ~3–5 nm. These randomly oriented graphitic crystals are marked in Figure 1. The existence of amorphous carbon material was clearly suggested by the diffraction patterns of the sample films, shown in Figure 1. Furthermore, the existence of randomly oriented graphite nano-clusters on the (100) plane can also be observed using the diffraction pattern. It was observed from the diffraction pattern and the HRTEM image that the a-Carbon sample films were a combination of graphitic nano-clusters and amorphous carbon.

**Figure 2:**
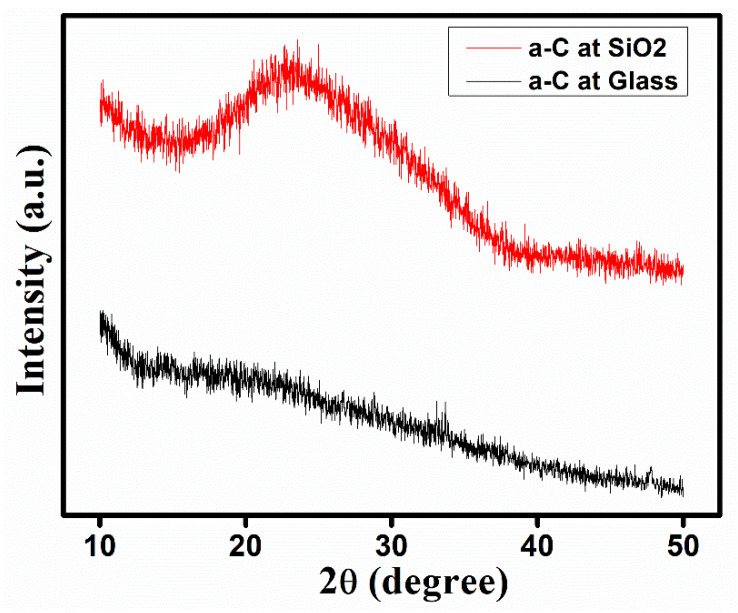
XRD pattern of a-Carbon sample films fabricated on glass and SiO_2_ substrates.

With the help of X-ray diffraction (XRD), the crystalline behavior of the material was investigated. Figure 2 illustrates the XRD patterns for both the a-Carbons grown on glass and SiO_2_ substrates. The XRD spectrum of the a-Carbon on glass showed a completely amorphous quality, while the a-Carbon on SiO_2_ showed a large carbon hump around ~25°, which was attributed to the 002 peak of the graphite plane. As our specimen was amorphous, some randomly oriented graphitic crystal structures with a size of 3–5 nm were also found, which are explained in Figure 1. These graphitic-like randomly oriented crystals were more obvious in the SiO_2_ specimen compared to the glass substrate specimen. This is why the XRD pattern of a-Carbon on glass showed as being amorphous while the a-Carbon on the SiO_2_ substrate showed a graphitic carbon hump around ~25°.

**Figure 3:**
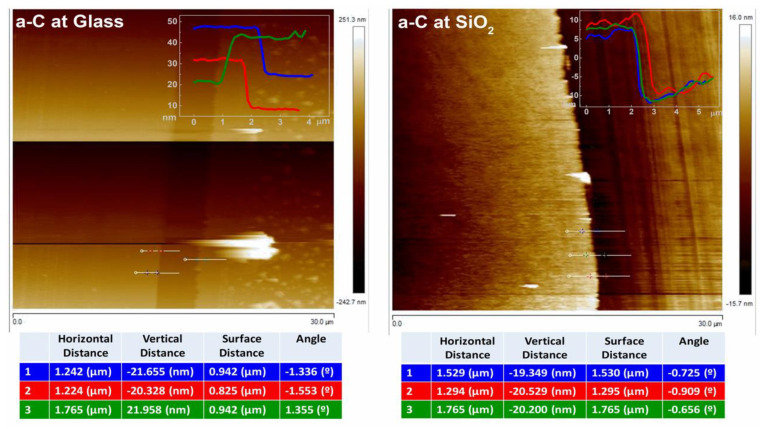
AFM surface morphology spectra and thickness plots for a-Carbon on glass and SiO_2_ substrates.

To observe the substrate effect on the growth and structural properties of these thin film samples, we performed AFM tests for both samples. Figure 3 shows the different surface morphologies and the similar thickness of the samples on both the glass and SiO_2_ substrates. The average thickness of the glass and SiO_2_ substrate samples were observed as 21 ± 1 nm and 20 ± 1 nm, respectively. A very small difference in thicknesses was observed for both samples, but the surface morphologies and the structural properties varied a great deal. Thus, it could be estimated that differences in the substrates could play important roles in tuning the structural properties and growing different disordered samples. This structural disorder of the amorphous thin films mainly contributes to tuning the MR properties.

**Figure 4:**
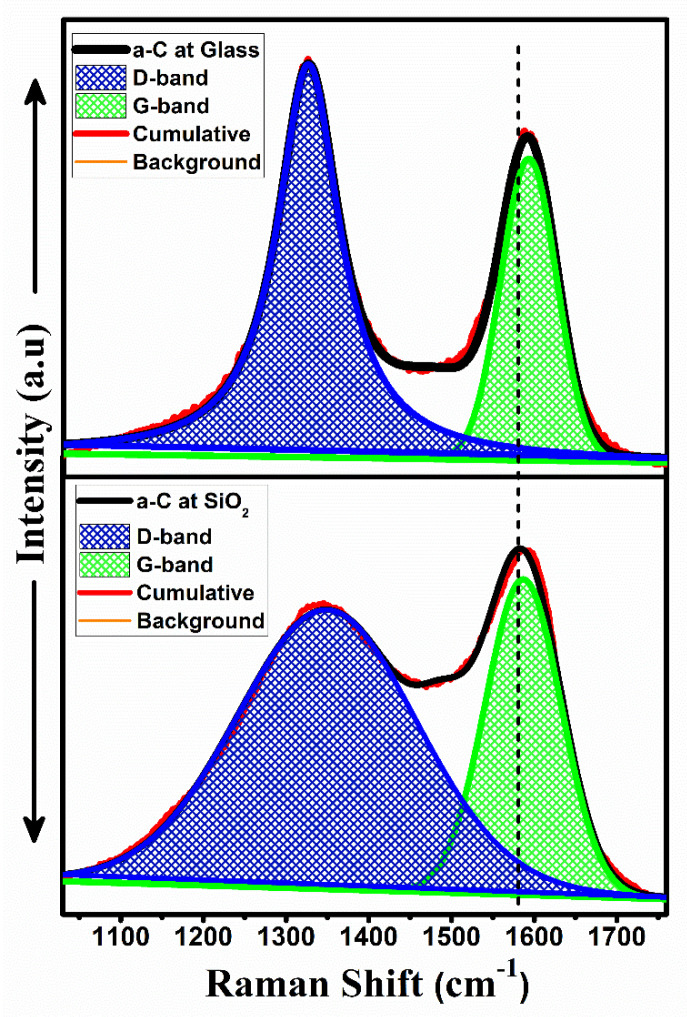
Raman bands of sample a-Carbon films on glass and SiO_2_ substrates.

Figure 4 shows the Raman spectra of a disordered a-Carbon samples fabricated on glass and SiO_2_ substrates. It has been observed that both major G-band and D-band peaks were found at ~1586 cm^−1^ and ~1349 cm^−1^, respectively, for the a-C on the SiO_2_ specimen, while, on the other hand, for the glass substrate sample, G-band and D-band peaks were found at ~1602 cm^−1^ and ~1350 cm^−1^, respectively. A large shift in the G-band position was observed from 1586 cm^−1^ to ~1602 cm^−1^. Since the D-band is considered to be the breathing mode of A_1g_ symmetry, and it requires defects for activation, therefore, it is further leading to the presence of defects and is a major focus in observing this band in our study. In a-Carbon, the peak intensity I_D_/I_G_ ratio can be related with the C(*sp*^2^) carbon content ratio to estimate the structural disorder [28,29]. The I_D_/I_G_ ratio was found to be increased from 1.1 ± 0.1 to 2.4 ± 0.2 in the thin films with an increase in *sp*^2^ carbon content. From the Lorentzian fitting function, the FWHM of the D-band peak was estimated to be 86 cm^−1^ and 257 cm^−1^ for the glass and SiO_2_ substrates, respectively. As this D-bands represents the disorder in a structure, the SiO_2_ substrate sample was observed to be a more disordered structure compared to the glass substrate sample.

**Figure 5:**
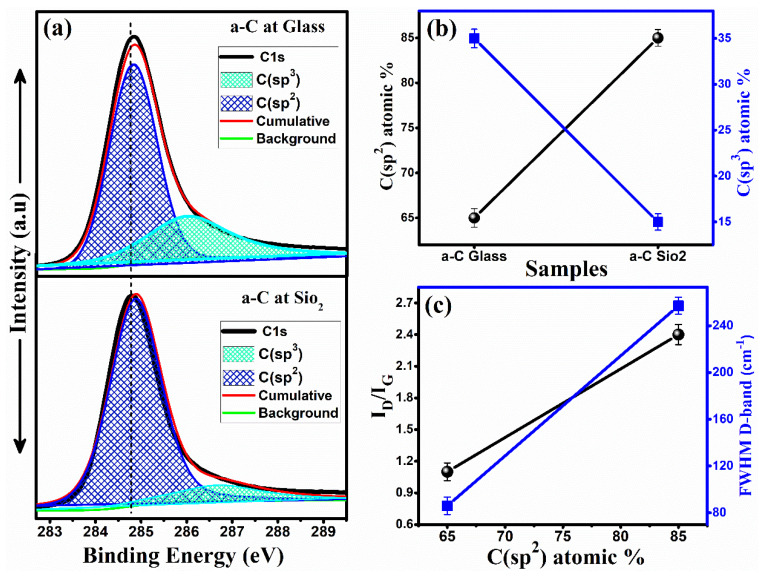
XPS spectra of sample a-Carbon (**a**) on a glass and SiO_2_ substrate; (**b**) C(*sp*^2^) and C(*sp*^3^) ratio; (**c**) I_D_/I_G_ ratio vs FWHM of the D-band.

The development of *sp*^2^ and *sp*^3^ bonding appears due to the difference in hybridization of existing carbon atoms. The graphitic rings in the form of π and π* bands give rise to *sp*^2^ hybridized atoms and the aliphatic chains give rise to the *sp*^3^ part [29]. From Figure 5, it is clear that typical XPS decomposition peaks of sample a-Carbon specimens were observed at ~284.8 and ~286.5 eV, corresponding to C(*sp*^2^) and C(*sp*^3^) bonds, respectively. It was observed that the amorphous carbon structure was mostly made of C(*sp*^3^) and C(*sp*^2^) contents, and C(*sp*^2^) was dominant with an 85% atomic ratio for the a-Carbon on the SiO_2_ substrate, while, on the other hand, the C(*sp*^2^) content ratio for the glass substrate sample was estimated to be 65%. Thus, in combination, the a-Carbon sample films were made of C(*sp*^3^) and C(*sp*^2^) cluster contents.

As the carbon material consisted of a majority of *sp*^2^ clusters and are good conductors, while the carbon material consisted of a majority of *sp*^3^ clusters and are not good conductors [26], the samples with a larger C(*sp*^2^)/C(*sp*^3^) ratio will have excellent conduction properties. We observed the magnitude for the *sp*^2^ cluster ratio of 85% for the a-Carbon on the SiO_2_ specimen, as shown in Figure 5b. Furthermore, the I_D_/I_G_ and FWHM of the D-band as a function of C(*sp*^2^) content ratio is also presented in Figure 5c. It can be observed that both the I_D_/I_G_ and FWHM of the D-band increased with an increase in the C(*sp*^2^) content ratio.

The FWHM of the D-band and I_D_/I_G_ ratio of the Raman spectrum are interrelated with the C(*sp*^2^) ratio. Therefore, our experimental Raman results are in better agreement with the XPS results. The higher I_D_/I_G_ ratio resulted in disordered structures and this ratio increased with a rise in FWHM of the D-band peak, which further contributed to the tuning of the magnetoresistance properties.

### 3.2. Magnetoresistance Properties

**Figure 6:**
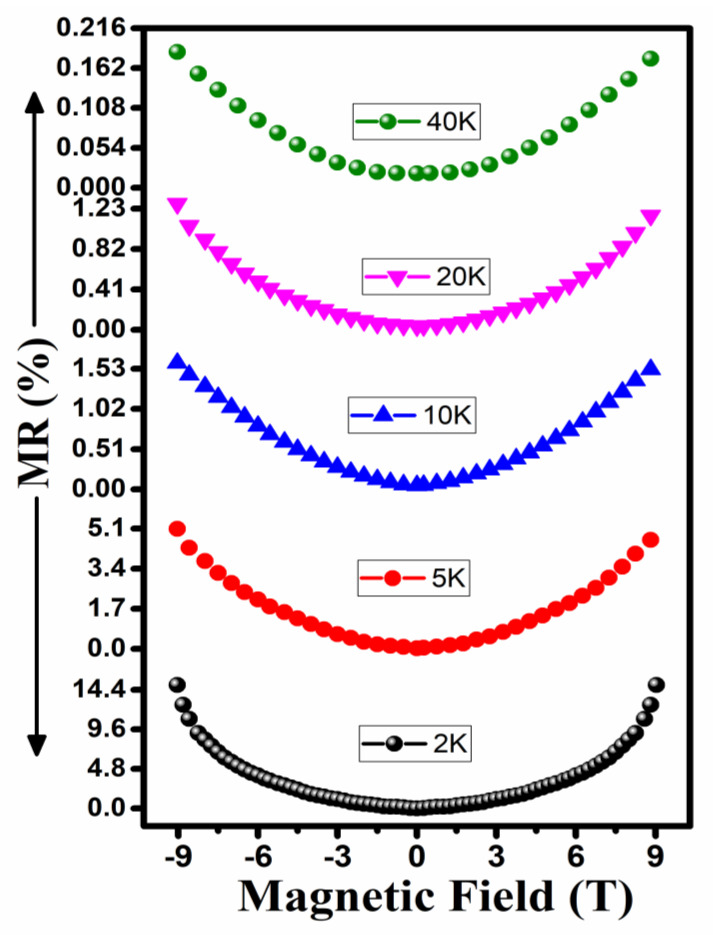
MR-B curves for temperature ranges from 2 K to 40 K.

The magnetoresistance features of the a-Carbon thin films have been examined in a temperature range of 2 K to 40 K under a magnetic field ranging from −9 T to 9 T. MR was calculated with the help of a varying magnetic field according to the following definition [30].

(1) $$MR\;\left(\%\right)=\frac{\left(R_{B}-R_{0}\right)}{R_{0}}\times 100$$

where $R_{0}$ and $R_{B}$ are the resistances of the specimens without a magnetic field and at definite field *B*, respectively. The MR-B curves of the undoped a-Carbon samples were measured from −9 T to 9 T for magnetic fields at various temperatures, ranging from 2 K to 40 K, as shown in Figure 6. Maximum MRs of ~15% and ~12% were investigated under a 9 T field and a measuring temperature of 2 K for the thin film samples deposited on glass and SiO_2_ substrates, respectively. The magnitude of MR was found to be decreased through an increase in the measurement temperature and also an MR-B shape change was observed for both specimens.

Since, we investigated the Raman and XPS results and it was observed that disorder increased with the use of SiO_2_ substrate instead of a glass substrate. As a result, these C(*sp*^2^) carbon clusters were increased in the specimen and helped to increase the grain boundaries at the film surface. So, the grain boundaries will cause further defects. The edges for the crystals were considered to behave as defects boundaries; thus, a very high MR magnitude was examined when surface disorder arises in a-Carbon thin film samples.

**Figure 7:**
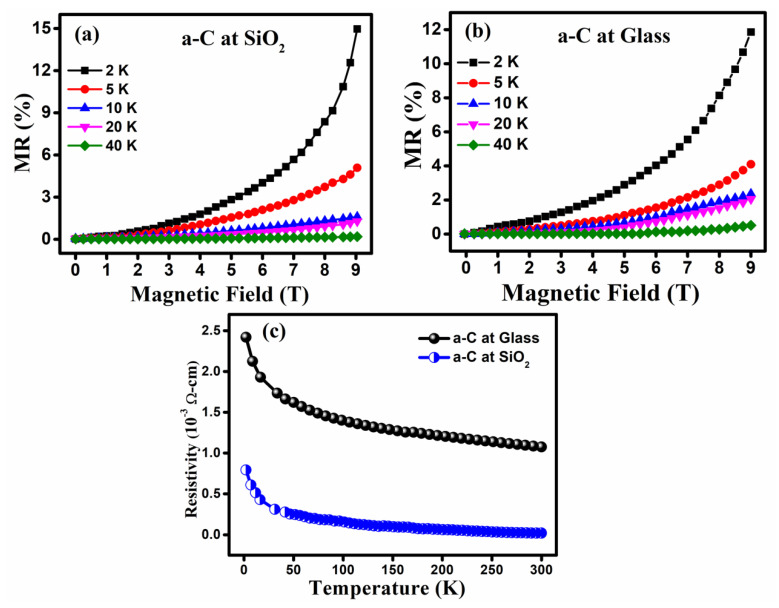
MR vs. magnetic field (T) (**a**) for a-Carbon on SiO_2_ substrate; (**b**) a-Carbon on glass substrate; (**c**) resistivity vs. temperature curves.

Figure 7a,b illustrates the MR-B curves for thin film specimens, which were located in the positive magnetic field range. When we selected different temperature ranges, from 2 K to 40 K, the same trend for MR, due to the magnetic field, was obtained. At a low measurement temperature of 2 K, a higher MR magnitude is observed, and the MR value suddenly decreased with a rise in the measurement temperature. We have observed, in both specimens, that the MR disappeared. Hence, it is clear that MR is only a low-temperature phenomenon for pulsed laser deposited thin films. MR is the change in the resistance phenomenon; Figure 7c shows that the resistivity plot of the thin films decreased abruptly by increasing the measurement temperature. For a higher temperature range, the change in resistivity is very small.

The scattering phenomenon occurs at the nanocrystalline grains boundaries, which are engrained in an amorphous system, and such grain boundaries act as potential (δ) barriers [31]. A similar phenomenon occurred for our a-Carbon thin films, in which randomly oriented graphitic nanocrystallites were embedded in the amorphous carbon matrix. Thus, the a-Carbon sample thin films had a combination of both amorphous and randomly orientated nanometer-sized graphitic clusters on the surface. The transport conduction occurring to these amorphous thin films might be a response of these graphitic clusters.

If we compare the nano-dimensioned material to the bulk materials, then the electrical resistivity is mainly dependent on the surface and grain boundaries, as suggested by Mayadas and Shatzkes [30]. This phenomenon can estimate the resistivity variation trends for the embedded nanocrystalline amorphous carbon sample thin films [31]. The scattering phenomenon for electrons at the boundaries cannot be considered as an exclusive mechanism. As the tunneling effect of electrons among the *sp*^2^ clusters can contribute to the conduction phenomenon, and, moreover, to the MR of the sample films, we will investigate this in the future. The substrate effect for such a-Carbon sample films showed a significant role in structural and magnetotransport characteristics when fabricated under different growth conditions, and may demand more attentions from scientists.

## 4. Conclusions

In this paper, the PLD technique was used to fabricate amorphous carbon thin films on SiO_2_ and glass substrates. We have investigated the variations in the structural and MR properties due to the substrate effect. The structural disorder in the thin films was investigated using Raman and XPS techniques. The I_D_/I_G_ ratio was observed to be 1.1 to 2.4 for the amorphous carbon thin films on the glass and SiO_2_ substrates, respectively. For the SiO_2_ substrate thin film specimen, the C(*sp*^2^) atomic ratio value was observed to be ~85. A maximum magnetoresistance of 15% at 9 T and 2 K was estimated for the SiO_2_ substrate sample thin film. The MR phenomenon appeared only at lower temperatures, whereas for high temperatures, above 40 K, MR disappeared very quickly. In comparison with the glass substrate sample thin films, the SiO_2_ substrate film was more disordered and had a higher magnitude of magnetoresistance, which was ascribed to the increasing degree of disorder.

## Data Availability

Not applicable.
